# Training Multitasking Abilities in Cognitively Healthy Older Adults: Training Gains and Transfer Effects of a Digital Pilot Randomized Controlled Trial

**DOI:** 10.1002/alz70860_100616

**Published:** 2025-12-23

**Authors:** Kayri K. Bertolotta, Michelle Hernandez, Maya Gal, Daniel Ben‐Eliezer, Andrei Teodorescu, Michal S Beeri, Seonjoo Lee, Sabrina Simoes, Sylvie Belleville, Benjamin M. Hampstead, Daniel Gopher, Yaakov Stern, Sharon Sanz Simon

**Affiliations:** ^1^ Fordham University, New York, NY, USA; ^2^ Columbia University Irving Medical Center, New York, NY, USA; ^3^ Columbia University, New York, NY, USA; ^4^ Technion ‐ Israel Institute of Technology, Haifa, Israel; ^5^ Herbert and Jacqueline Krieger Klein Alzheimer's Research Center at Rutgers Brain Health Institute, New Brunswick, NJ, USA; ^6^ New York State Psychiatric Institute, New York, NY, USA; ^7^ Taub Institute for Research in Alzheimer's Disease and the Aging Brain, Columbia University, New York, NY, USA; ^8^ Institut Universitaire de Gériatrie de Montréal, Montréal, QC, Canada; ^9^ University of Michigan, Ann Arbor, MI, USA; ^10^ Columbia University Vagelos Collège of Physicians and Surgeons, New York, NY, USA; ^11^ New Jersey Medical School, Rutgers University, Newark, NJ, USA

## Abstract

**Background:**

Developing efficient cognitive training programs for the older population is a major public health goal due to its potential benefits on cognition and quality of life. A promising training approach is emphasis change, which can benefit executive/attention control and multitasking abilities. The aim of this digital pilot randomized controlled trial was to assess the effects of the emphasis change using a new web‐based platform that simulates real‐life multitasking demands, the Breakfast Game.

**Method:**

A community‐based sample of 38 cognitively healthy participants (M = 65.8, SD = 3.6), highly educated (M = 16.4, SD = 2.0) were randomized between two conditions: 1) Emphasis Change (EC): participants were instructed to place particular emphasis on specific aspects of the game; and 2) Active Control (AC) – gameplay with standard instructions. Participants underwent 13 online sessions using the Breakfast Game. Each session lasted one hour and occurred 3 times a week at participants’ homes. Five out of the 13 sessions were supervised via videoconference. Participants completed a neuropsychological assessment via videoconference at baseline and after the intervention (clinicaltrials.gov ID: NCT05506852).

**Result:**

At baseline, participants from both conditions showed similar levels of cognitive performance and computer literacy (*p* > .05). The trial presented high adherence (94.5%) and retention rates (89%), with a loss of 2 participants per group. Both groups show a learning curve, with time effects in the game outcomes (*p* < .05). A time‐by‐group interaction (*p* = .04) was observed for one game accuracy measure (e.g., range of stop times), indicating greater training gain in the EC group. Regarding the transfer effects, there were time‐by‐group interactions (*p* = .02; *p* = .03) in the primary outcome (Alphanumeric Task), showing greater improvement in the EC in modulating their attention allocation. There was a modest effect in the secondary transfer outcome, the executive functions composite score (*p* = .03), mainly driven by working memory and divided attention tasks. There were no changes in the self‐efficacy and mood measures.

**Conclusion:**

Online emphasis change training using the Breakfast Game is feasible with modest effects and promising benefits on divided attention/executive control in older adults. Further research should improve the features of the Breakfast Game and clarify the dose‐response relationship.

